# Balancing Robustness and Evolvability

**DOI:** 10.1371/journal.pbio.0040428

**Published:** 2006-12-12

**Authors:** Richard E Lenski, Jeffrey E Barrick, Charles Ofria

## Abstract

Can a single unifying mathematical framework help to explain robustness - the ability of organisms to persist in the face of changing conditions - at all biological scales, from biochemical to ecological?

One of the most important features of biology is the ability of organisms to persist in the face of changing conditions. Consider the remarkable fact that every organism alive today is the product of billions of generations in which its progenitors, without fail, managed to produce progeny that survived to reproduce. To achieve this consistency, organisms must have a balance between robustness and evolvability, that is, between resisting and allowing change in their own internal states [1–3]. Moreover, they must achieve this balance on multiple time scales, including physiological responses to changes over an individual life and evolutionary responses, in which a population of genomes continually updates its encoded information about past environments and how future generations should respond given that record.

Examples of robust biological systems are found at many scales, from biochemical to ecological. At each scale, robustness may reflect the properties of individual elements or, alternatively, the dynamic feedbacks between interacting elements. The expression of some metabolic function, for example, may be robust in the face of temperature change, because an enzyme maintains its shape and specificity across a range of temperatures or because an interconnected network of reactions sustains the supply of product, even when some enzyme fails. A genome may be robust because it encodes proofreading and repair systems that reduce replication errors or because it is organized such that many mutations have little effect on its phenotype. An ecosystem might be robust if it resists the extinction of some keystone species or, if extinction does occur, because surviving species can compensate over physiological, demographic, or evolutionary time scales.

One important question is whether there exists a single unifying mathematical framework that can encompass such diverse examples of biological robustness. Might new insights come from such a conceptual unification, or will future understanding require detailed analyses of specific cases? Across the different scales, recurring mechanisms for achieving robustness—including redundancy of component parts and negative feedbacks—might serve as organizing principles. Yet, similarities in mechanism could mask important differences in the evolutionary origins of those mechanisms. At the level of genes in genomes or of cells in multicellular organisms, it is reasonable to suggest that redundancy evolved by natural selection to maintain some functional capacity in the face of perturbation [4]. But whereas species redundancy could also be critical for robustness of ecosystem functions, differences in redundancy might be an emergent property rather than an ecosystem-level adaptation, because selection generally acts at lower levels (but see [5] for another view).

And if robustness has evolved to maintain performance, what prevents systems from becoming ever more robust? We will focus on genomic robustness to mutations, because it provides a concrete example, although many ideas are speculative and much work is needed to formalize and test them. Two mechanisms that could make a genome more robust are genetic redundancy, so that many otherwise deleterious mutations are masked, and proofreading during replication, so that fewer mutations occur. Redundancy imposes a cost of replicating the additional gene copies [6], whereas proofreading entails costs of encoding and expressing that function [7].

Mutational robustness can also arise in more subtle ways. Populations evolving at high mutation rates may settle in regions of genotypic space where mutations are less deleterious, on average, than those regions that attract populations that experience low mutation rates. The idea is that evolution at low mutation rates favors populations that achieve high fitness peaks, even if they are surrounded by steep cliffs, because mutations that push progeny off those cliffs are rare. By contrast, at high mutation rates, most offspring carry mutations, and selection favors populations that find lower fitness peaks surrounded by less precipitous mutational chasms. Experiments with digital organisms (self-replicating computer programs) provide direct support for “survival of the flattest” at high mutation rates [8]. RNA viruses also have very high mutation rates, and a recent experiment implicated the importance of mutational robustness for them, in this case, by showing the loss of robustness in viruses that evolved at high multiplicities of infection, where co-infecting particles guaranteed redundancy and allowed their native robustness to decay [9].

But generalizing to other organisms presents some difficulties. The strength of selection for robustness should be weaker in larger genomes if the advantage to a mutation that increases robustness locally is correspondingly smaller. According to one alternate hypothesis, mutational robustness is not so much a directly evolved property as it is a correlated benefit of selection for robustness in the face of variable environments [10]. The essential ideas here are that environmental change is a pervasive feature of nature, and those physiological mechanisms that allow organisms to adjust to changing environments, such as by regulating gene expression, will also compensate for the effects of many mutations [3]. Robustness might also evolve to minimize internal noise in biochemical systems. The genetic code itself, once viewed as a frozen accident from the early history of life, has been shown to be remarkably well designed for minimizing the production of proteins that, owing to translational errors, have the amino acids most likely to disrupt protein function [11]. Individual proteins, too, have been strongly selected for robustness to translational errors [12].

Two recent studies with evolving computational systems have shown, unexpectedly, that sexual reproduction promotes the evolution of mutational robustness [13,14]. The evolutionary value of sex is a fascinating old problem. According to one hypothesis, the advantage of sex depends on negative interactions between deleterious mutations, such that two mutations combined tend to be worse, on average, than expected from their individual effects [15]. In that case, sex helps to purge them and provides a kind of robustness to multiple mutations. But these new studies found that sexual populations became more robust, on average, to the effects of single mutations, even though they evolved at the same mutation rate as asexual controls. Sex bombards genomes with mutant alleles that arose in other genetic backgrounds, which evidently promotes a kind of “survival of the flattest” similar to that seen at high mutation rates.

Another important issue revolves around the tension between robustness and evolvability. Are genomes that are more robust to mutations less evolvable in the face of changing environments? In other words, does canalizing the phenotype to minimize perturbations—including biochemical and environmental as well as mutational—lead to an evolutionary conservatism that inhibits the discovery of new adaptive solutions? Some mechanisms of robustness, such as proofreading and repair, must inhibit evolvability because they reduce the production of new beneficial mutations. But are robustness and evolvability inversely correlated more generally? In the case of redundancy, the presence of multiple gene copies might mask the beneficial effects of some new mutations, thus suppressing evolvability. But redundancy can also promote adaptation by allowing duplicated genes to evolve distinct functions [16,17].

Evolving populations can also become robust by finding regions of genotypic space that are flat because they contain a high proportion of neutral mutations [18]. As shown schematically for RNA secondary structures in Figure 1, the resulting neutral network might provide evolutionary paths to new adaptations by random drift, in effect allowing populations to search wider regions of genotypic space for rare beneficial mutations [19]. If so, robustness and evolvability might again be positively, rather than negatively, correlated. However, deleterious mutations can also serve as stepping stones to adaptations [20]. Although deleterious mutations tend to be removed by selection and have shorter half-lives than neutral mutations, they are not instantly eliminated. Moreover, deleterious mutations may lead to genetic neighborhoods that are more promising, from the perspective of adaptation, than neutral mutations. In other words, neutral mutations are neutral precisely because they are isolated from important phenotypes, whereas deleterious ones must be connected to phenotypes that matter for fitness. It is unclear, therefore, whether neutral or deleterious mutations are more important for evolvability, and whether robustness associated with increased neutrality will promote or impede evolvability.

**Figure 1:**
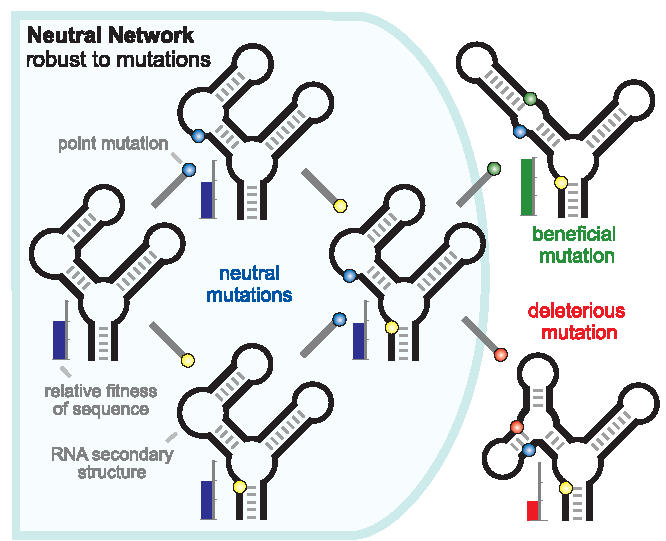
A Neutral Network of Four RNA Secondary Structures, with One Member Connected to Two Sequences outside the Network, One with Lower, and One with Higher Fitness Colored positions show mutations, whereas the scale to the left of each sequence shows its relative fitness. (Figure credit: Jeffrey E. Barrick, Michigan State University, East Lansing, Michigan, United States)

Theoretical population genetics has historically emphasized models with one or two loci, whereas quantitative genetics has relied on a sort of statistical mechanics that ignores underlying detail. Richer mathematical representations of genotypic spaces and fitness landscapes may be required to understand the balance between robustness and evolvability. Meanwhile, empiricists must push ahead to obtain data about evolvability and robustness. Experimental evolution, in which populations are monitored while they evolve under defined conditions, offers the potential to observe changes in these properties as a function of environmental and genetic manipulations [21]. For example, one could ask how robustness and evolvability change depending on whether evolution occurs in constant or variable environments [22]. Or one might instead manipulate an organism's regulatory networks to investigate how that affects these properties. As new insights are achieved into the tension between genomic robustness and evolvability, perhaps the findings can inform investigations into robustness at other levels, from cells to ecosystems, as biologists seek to understand the constancy of change.
